# COGNITIVE, PHYSICAL, AND PSYCHOLOGICAL FUNCTION AND QUALITY OF LIFE IN PATIENTS WITH STROKE—A NETWORK ANALYSIS

**DOI:** 10.1093/geroni/igad104.0658

**Published:** 2023-12-21

## Abstract

This study aimed to identify the dynamic associations across cognitive, physical and psychological function, and quality of life using network analysis among Chinese patients with acute ischemic stroke (AIS). We conducted a cross-sectional study in 2021 that included a total of 636 patients with AIS from three stroke centers in Shanghai, Nanjing, and Linyi, China. Participants completed a complex battery of measures of cognitive function (Montreal Cognitive Assessment, MoCA), physical function (Barthel Index, Modified Rankin Scale), psychological function (Epidemiological Studies Depression Scale, CES-D) and quality of life (short version of Stroke-specific Quality of life scale, SS-QOL). We used the Gaussian Graphical Modeling to estimate the network structure. Cognitive and physical function were important prerequisites to quality of life. Specifically, the most central nodes (strength) in the network were quality of life, attention (cognitive domain), transferring to a chair, caring perineum/cloth at toilet and walking (physical domain). Cognitive function, particularly visuospatial /executive function and attention, and physical (dressing, feeding and drinking, up and downstairs and transferring to a chair) were positively related to quality of life; whereas depressive symptoms and disability were negatively related to quality of life. Factors contributing to quality of life are complex in patients with AIS. Attention, visuospatial /executive function from cognitive function and transferring, caring at toilet and walking from physical function were central nodes to the quality of life network. Interventions that target cognitive and physical function may have a potential to improve quality of life for stroke patients.

